# 

**Published:** 1888-07

**Authors:** 


### PROGRAMME FOR THE JOINT SESSION OF THE AMERICAN AND SOUTHERN DENTAL ASSOCIATIONS.

### OFFICERS AMERICAN DENTAL ASSOCIATION.

Frank Abbott, President; C. R. Butler, First Vice-President; T. S. Waters, Second Vice-President; F. A. Levy, Corresponding Secretary; Geo. H. Cushing, Recording Secretary; Geo. W. Keely, Treasurer.

Executive Committee: A. W. Harlan, Chairman; S. H. Guilford, Secretary.

First Division—Committee of Arrangements: A. W. Harlan, W. C. Wardlaw, L. D. Shepard.

Second Division—On Credentials, Ethics and Auditing Accounts: A. O. Hunt, A. M. Dudley, S. G. Perry.

Third Division—On Voluntary Essays: S. H. Guilford, E. T. Darby, A. H. Thompson.

### OFFICERS SOUTHERN DENTAL ASSOCIATION.

B. H. Catching, President; *J. H. Prewitt, First Vice-President; W. N. Morrison, Second Vice-President; J. Hall Moore, Third Vice-President; J. Y. Crawford, Corresponding Secretary; L. P. Dotterer, Recording Secretary, H. A. Lowrance, Treasurer.

Executive Committee: C. G. Edwards, Chairman; B. O. Doyle, W. McL. Dancy.

### TUESDAY MORNING, 9 A. M.

Separate meetings of the two Associations for the payment of dues, the receiving of credentials, and the transaction of routine business.

### AFTERNOON, 2.30 P. M.

Meeting of the different joint Committees for the examination of the papers to be read by them.

### EVENING, 7.30 P. M.

As the joint session is only for scientific and social purposes, nothing but professional subjects will be discussed or acted upon.

Meeting of the joint session to be presided over by Frank Abbott and B. H. Catching.

The President of the Southern Dental Association will call the meeting to order, announce the prayer, and then introduce the President of the American Dental Association, who will deliver his address; after which the latter will introduce the President of the Southern Dental Association, who will then present his address. The President of the American Dental Association will continue in the chair for the remainder of that evening.

President Abbott's address; President Catching's address; discussion on the same. Reports of joint Committees and discussions thereon. Announcements from the Chair. Adjournment.

### WEDNESDAY MORNING, 9 A. M.

Meeting of the joint session presided over by B. H. Catching. Reports of joint Committees and discussions of same. Announcements. Adjournment, 1 P.M.

### AFTERNOON.

To be devoted to the work of the joint Committees or to business meetings of the Associations, if such should be necessary, or to Clinics.

### EVENING, 7.30 P. M.

Meeting of joint sessions, presided over by Frank Abbott. Reports of joint Committees and discussions. Announcements. Adjournment.

### THURSDAY MORNING, 9 A. M.

Meeting of joint session, presided over by B. H. Catching. Reports of joint Committees and discussions. Announcements. Adjournment, 1 P. M.

### AFTERNOON.

Committee work, or business meetings, or Clinics.

EVENING, 7.30 P. M.

Meeting of joint session, presided over by Frank Abbott. Reports of joint Committees and discussion. Announcements. Adjournment.

FRIDAY MORNING, 9 A. M.

Meeting of the joint session presided over by B. H. Catching. Reports of joint Committees and discussions. Announcements. Adjournment, 1 P. M.

AFTERNOON, 3 P. M.

Separate meetings of the two Associations for the selection of place of next meeting, the election of officers, and the transaction of such other business as may come before them.

EVENING, 7.30 P. M.

Meeting of joint session, presided over by Frank Abbott. Reports of joint Committees and discussion. Announcements. Final adjournment of joint session for discussion.

SATURDAY MORNING, 8.30 A. M. to 1.30 P. M.

Clinics.

AFTERNOON, 3 P. M.

Separate meetings of the two Associations for closing business.

The joint Committees will be called in the following order, and any Committee failing to respond will be passed and not again called until all the others have been.

The reports of these Committees will be written and offered by the Chairmen, and will specify the papers to be presented and the order in which they shall be read, and such subjects for discussion or such suggestions as they may wish to bring to the attention of the joint session.

All papers to be read before the joint session, except the two Presidents' addresses, must be placed in the hands of the Chairmen of the appropriate joint Committees, who will examine them and report those only they deem worthy of presentation to the joint session.

The following Rules of order will govern this joint session.

No member of either Association shall be entitled to the floor unless he is in good standing and his dues are fully paid.

No person shall speak more than twice upon the same subject, nor more than ten minutes in all, unless consent is given by a majority vote of the joint session.

No one shall be permitted to address the meeting before giving his name and residence, which shall be distinctly announced from the Chair.

When a paper has been read it shall at once be handed to the Secretary of the Association from which it came.

Any paper or report to be entitled to publication in the transactions must be placed in the hands of the Publication Committee by the 15th of September, 1888, and must be so prepared that the proof-sheets furnished the author shall be returned to the Committee without material alteration or addition.

*Roberts' Rules of Order* shall be the authority governing this meeting, if any is needed more than is embodied in the foregoing rules.

Attention is called to the first, second and third Rules of Order, page 12.

If gentlemen addressing the Association will give particular heed to these requirements, and will, when they rise to speak, announce in a loud and clear voice their name and residence, it will tend greatly to facilitate business.

A book for the registration of members will be found at the entrance of the place of meeting, and all members are requested to register their names and hotel addresses at the earliest moment possible.

The dental depots will be promptly closed at the hour for the opening of each session, and will remain closed until the adjournment of the same.—*Sonthern Dental Journal.*

### *To Readers and Correspondents!*

Communications intended for publication, and exchange journals and papers should be addressed to the EDITOR OF THE AMERICAN JOURNAL OF DENTAL SCIENCE, 845 N. Eutaw St., Baltimore, Md. All letters relating to the business of the JOURNAL, or containing cash remittances, to be directed to Messrs. SNOWDEN & COWMAN. Articles for publication should be received by the editor at least three weeks previous to the first month on which the number in which it is designed for them to appear, is issued.

☞ The AMERICAN JOURNAL OF DENTAL SCIENCE will contain fifty pages of reading matter in each number, making a volume of about

*Six Hundred* pages,

EXCLUSIVE OF ADVERTISEMENTS.

